# microRNA-29 negatively regulates EMT regulator N-myc interactor in breast cancer

**DOI:** 10.1186/1476-4598-13-200

**Published:** 2014-08-29

**Authors:** Jack W Rostas, Hawley C Pruitt, Brandon J Metge, Aparna Mitra, Sarah K Bailey, Sejong Bae, Karan P Singh, Daniel J Devine, Donna L Dyess, William O Richards, J Allan Tucker, Lalita A Shevde, Rajeev S Samant

**Affiliations:** Department of Pathology, University of Alabama at Birmingham, WTI-320E, 1824 6th avenue South, Birmingham, AL 35294 USA; Department of Surgery, University of South Alabama, Mobile, AL USA; Mitchell Cancer Institute, University of South Alabama, Mobile, AL USA; BBSF-Comprehensive Cancer Center, University of Alabama at Birmingham, Birmingham, AL USA; Department of Pathology, University of South Alabama, Mobile, AL USA; Comprehensive Cancer Center, University of Alabama at Birmingham, Birmingham, AL USA

**Keywords:** N-Myc interactor, EMT, Breast cancer, miR-29

## Abstract

**Background:**

N-Myc Interactor is an inducible protein whose expression is compromised in advanced stage breast cancer. Downregulation of NMI, a gatekeeper of epithelial phenotype, in breast tumors promotes mesenchymal, invasive and metastatic phenotype of the cancer cells. Thus the mechanisms that regulate expression of NMI are of potential interest for understanding the etiology of breast tumor progression and metastasis.

**Method:**

Web based prediction algorithms were used to identify miRNAs that potentially target the NMI transcript. Luciferase reporter assays and western blot analysis were used to confirm the ability of miR-29 to target NMI. Quantitive-RT-PCRs were used to examine levels of miR29 and NMI from cell line and patient specimen derived RNA. The functional impact of miR-29 on EMT phenotype was evaluated using transwell migration as well as monitoring 3D matrigel growth morphology. Anti-miRs were used to examine effects of reducing miR-29 levels from cells. Western blots were used to examine changes in GSK3β phosphorylation status. The impact on molecular attributes of EMT was evaluated using immunocytochemistry, qRT-PCRs as well as Western blot analyses.

**Results:**

Invasive, mesenchymal-like breast cancer cell lines showed increased levels of miR-29. Introduction of miR-29 into breast cancer cells (with robust level of NMI) resulted in decreased NMI expression and increased invasion, whereas treatment of cells with high miR-29 and low NMI levels with miR-29 antagonists increased NMI expression and decreased invasion. Assessment of 2D and 3D growth morphologies revealed an EMT promoting effect of miR-29. Analysis of mRNA of NMI and miR-29 from patient derived breast cancer tumors showed a strong, inverse relationship between the expression of NMI and the miR-29. Our studies also revealed that in the absence of NMI, miR-29 expression is upregulated due to unrestricted Wnt/β-catenin signaling resulting from inactivation of GSK3β.

**Conclusion:**

Aberrant miR-29 expression may account for reduced NMI expression in breast tumors and mesenchymal phenotype of cancer cells that promotes invasive growth. Reduction in NMI levels has a feed-forward impact on miR-29 levels.

## Introduction

N-myc interactor (NMI) is a cytokine (IL-2, IFNγ) inducible protein that interacts with several transcription factors such as STATs, cMYC, BRCA1, TIP60 and SOX10, all of which have known critical involvement in influencing tumor progression and stem-ness [1–6]. Thus, a range of signaling activities have been associated with NMI. NMI has been shown to augment STAT-mediated transcription in response to cytokines, specifically by mediating association of co-activator protein CBP/p300 with STAT1 or STAT5 [3]. It has also been implicated in negative regulation of cMyc driven hTERT transcription and positive regulation of a subset of SOX10 regulated transcripts [4, 5]. NMI shows compartmentalized location, either cytosolic or nuclear. For example, in C6 glioma cells nuclear NMI was more frequent in SOX10-expressing cells compared to cells that lacked SOX10 [5]. These different locations may impact its function. In response to cellular stresses, NMI is induced, and a fraction of NMI has been shown to translocate to the nucleus to stabilize ARF (INK4a/ARF), a tumor suppressor, and aid in stabilization of TP53 [7]. Disease relevance of NMI was highlighted in our previous observations which revealed that NMI expression is negatively correlated with stage and grade of breast tumors [8]. One of the mechanisms of NMI action in breast cancer was implied in its ability to negatively modulate transcriptional regulation of target genes by c-Myc. NMI facilitates formation of a tri-molecular complex that includes BRCA1, NMI and c-Myc [4]. Our quest to understand the details of functional relevance of NMI in cancer revealed that restoring expression of NMI in tumorigenic and invasive breast cancer and melanoma cell lines resulted in reduced growth of tumor xenografts in athymic mice [9]. Subsequent signaling studies revealed to us that NMI expression negatively regulates oncogenic Wnt/β-catenin signaling [9]. We also observed that silencing NMI expression from epithelial-like breast cancer cell lines induced molecular markers and morphological attributes of mesenchymal-like phenotype as well as promoted the invasive ability of these cells. Mechanistically, loss of NMI had negative impacts on STAT5-driven expression of TGFβ signaling repressor, SMAD7. This allowed for aberrant manifestation of TGFβ-driven epithelial-mesenchymal-transition (EMT) [8]. Based on this developing body of compelling reports, it is apparent that loss of NMI expression during tumor progression may prompt EMT and metastases. Thus the mechanisms that negatively regulate NMI expression are of potential interest to understand the etiology of metastatic progression of breast tumors and to identify potentially novel therapeutic targets.

While direct DNA mutations, translocation and deletions account for altered gene expression in cancer, epigenetic regulation, such as regulatory effects of non-coding RNA, specifically miRNA, have been found to be of equal importance in the development and propagation of many cancers [10–12]. MicroRNAs regulate gene expression *via* transcriptional modification and/or inhibition of translation [13, 14]. While much is known about specific mutations leading to an increased risk of developing breast cancer, similar roles for miRNAs are still being progressively uncovered [15–17]. Here we report our investigations that report for the first time, that NMI is a novel target of microRNA 29 (miR-29). Interestingly, we also observe that absence of NMI prompts a feed forward increase in miR-29 levels due to inactivation of GSK3β activity. Cumulatively, our studies reveal a novel inverse regulatory relationship of NMI and miR-29 in breast cancer.

## Materials and methods

All the methods were carried out in accordance with the approved guidelines.

### Target prediction

The N-myc (and STAT) intereractor (NMI); gene ID: 9111, mature mRNA sequence (NM_004688.2) was obtained from the NCBI and queried by the miRNA target prediction sites TargetScan(Human) [http://www.targetscan.org/] [18, 19] and microRNA.org [http://www.microrna.org] (miRaNda algorithm) [20, 21] and miRDB [http://mirdb.org/miRDB] [22–24]. The predicted binding strengths and scores were obtained. Only the miRNAs common to at least 2 searches were considered for further investigations.

### Cell culture and reagents

Cells lines were obtained from the ATCC. MDA-MB-231 and MDA-MB-435 cells were grown in DMEM/F12 supplemented with 5% heat inactivated fetal bovine serum. MCF7 cells were grown in DMEM/F12 supplemented with 10% heat inactivated fetal bovine serum and insulin (10 μg/mL) (Sigma, St Louis, MO, USA). T47D cells were grown in RPMI 1640 supplemented with sodium pyruvate (1 μg/mL), 10% heat inactivated fetal bovine serum, and insulin (10 μg/ml).

#### Antibodies

NMI mouse monoclonal (Sigma-9D8) (Sigma, St Louis, MO, USA), GAPDH rabbit monoclonal (mAb#2118), GSK-3β rabbit monoclonal (mAb#9315), E-cadherin rabbit monoclonal (mAB#3195), Keratin 8/18 mouse (mAb#4546) and phospho-GSK-3β-Ser9 rabbit monoclonal mAb#9323 (Cell Signaling Technologies Inc., Boston, MA, USA) were used.

### Quantitative RT-PCR

Cells were grown to 70% confluence and total RNA was harvested with TRIzol^®^ reagent (Life Technologies, Carlsbad, CA) or the SurePrep™TrueTotal™RNA purification kit (Fisher Scientific, Pittsburgh, PA, USA). RNA was quantitated and assessed using spectrophotometry (NanoDrop Lite, Thermo Scientific, Wilmington, DE, USA) for 260/280 and 260/230 ratio. Mature microRNA levels were assessed with strand-specific reverse transcription followed by quantitative PCR (Applied Biosystems, Foster City, CA.

Total RNA was used to generate cDNA using primers specific to RNU6B (control) or hsa-miR-29 a or b. PCR was performed using both U6 (control) or hsa-miR-29a/b Taqman primer probes and Taqman Universal Master Mix, No Amperase UNG (Applied Biosystems). miR-29 miRNA levels were normalized to U6 levels.

To assess changes in mRNA, cDNA was generated from 1 μg total RNA using the High Capacity Reverse Transcription cDNA synthesis kit (Applied Biosystems, Foster City, CA). Subsequently, PCR was done with TaqMan primer probes specific to E-cadherin, Slug, Snail, Zeb1, or GAPDH. GAPDH was utilized as a normalization control.

### Plasmid constructs

Oligos designed to encompass hsa-miR-29 target site in mRNA of NMI

**5′**--CTCTGAATCTTCTTTGTTTCAAATGGTGCTGCATGTTTTCAACTAA ---**3′** and **3′**--AGCTTTAGTTGAAAACATGCAGCACCATTTGAAACAAAGAAGATTCAGAGAGCT---**5′** were annealed and cloned into HindIII and SacI sites of the pMIR-Report vector (Ambion, Austin, TX) to generate pMIR-REPORT29-NMI. For expressing precursor miRNAs, we used HmiR0119-MR04 for 29a or HmiR0120-MR04 for 29b and CmiR0001-MR04 as miRNA scrambled control. For miRNA inhibition, HmiR-AN0371-AM02 for 29a or HmiR-AN0373-AM02 for 29b were used. These precursor and miRNA inhibitor plasmids were purchased from GeneCopoeia™ (Rockville, MD, USA).

### Luciferase assay

pMIR-REPORT29-NMI (with miR-29A or miR-29B precursors (Applied Biosystems) was transfected in MCF7 cells (70% confluent) using siPORT™NeoFX™ (Life Technologies) in serum free medium as per the manufacturer’s instructions. After incubation at 37°C in 5% CO_2_ for 36 hr, cells were lysed using Reporter Lysis Buffer as per the manufacturer’s instructions (Promega, Madison, WI). The luminescence was assessed using GloMax^®^ 20/20 luminometer (Promega). To block miR-29 expression, cells were grown to 70% confluence and were transfected using Lipofectamine 2000 (Life Technologies) in serum free media with antagomirs for microRNA 29 (Exiqon, Woburn, MA, USA). After 48 hr protein was isolated for analysis. For Wnt/β-catenin reporter assays, transfections in T47D cells were done with 200 ng total DNA (Top-Flash, M50) using Lipofectamine 2000.

### Three-dimensional assay

3D cultures were grown following the protocol by Debnath *et al.* [8, 25, 26]. Briefly, eight well-chambered cover glass slides (Millipore, Billerica, MA) were placed on ice and coated with 50 μl of 3D Culture Matrix Basement Membrane Extract Reduced Growth Factor (phenol red free) from Trevigen (Gaithersburg, MD, USA). The slide was incubated for 30 min in 37°C incubator. Cells (1000/well, 36 hr post transient transfection of either precursor or antagonizing microRNA) were plated in growth media containing 2% reduced growth factor basement membrane extract (Trevigen, Gaithersburg, MD). Media was changed every 4 days and the morphology was documented digitally using a Nikon Eclipse Ti-U microscope (Nikon, Tokyo, Japan) using the 20 × objective.

### Immunocytochemistry

Cells were seeded at 100,000 cells per well in a 6 well plate with 12 mm round poly-lysine coated glass coverslips. Cells were washed with PBS and then fixed for 30 minutes at room temperature in 4% paraformaldehyde. Coverslips were then washed with PBS and blocked with 5% BSA in PBS with 0.3% Triton X-100 for 1hr. E-cadherin antibody (Cell Signaling, Beverly, MA) was diluted 1:200 in 1% BSA in PBS with 0.3% Triton X-100 and the coverslips were incubated overnight. Coverslips were then incubated with anti-rabbit Alexa-Fluor 594 (Life Technologies, Grand Island, New York); diluted 1:400 in 1%BSA in PBS with 0.3% Triton X-100. Coverslips were washed with PBS and then were mounted onto glass slides using VectaSheild with DAPI (Vector Labs, Burlingame, CA). Images were captured using a Nikon Eclipse T*i*-U.

### Patient samples

After obtaining institutional review board approval (Approval #09-287; University of South Alabama), archived patient samples were obtained according to established institutional policies. Informed consent was obtained from all subjects. All specimens were coded and de-identified. The specimens comprised primary breast tumors and matched lymph node metastases obtained during surgical procedures which were immediately stored in liquid nitrogen. Tissue specimens were homogenized and RNA was extracted using QIAzol (Qiagen Inc., Valencia, CA). Mature microRNA levels were assessed using quantitative real-time PCR (miScript SYBR-green PCR kit, Qiagen) following cDNA synthesis (miScript II RT kit, Qiagen).

### Western blots

Cells were transfected with appropriate plasmid (or pre-miRNA or antagomiRs as appropriate) using Lipofectamine 2000 according to manufacturer’s instructions (Life Technologies). Cells were harvested 42 hr post transfection in NP-40 lysis buffer. The lysates (20 μg) were resolved on SDS-PAGE and transferred onto PVDF membranes. Immunoblots were developed using relevant primary and secondary antibodies as per the respective manufacturer’s instructions.

### Invasion assay

Invasion assays were conducted using 8 μM polyethylene terpthalate filters (BioCoat™ Matrigel Invasion Chambers, BD Pharmingen), as described earlier [27]. Cells (transfected with pre-miRs or antagomiRs as appropriate) were allowed to invade through matrigel coated filters for 16 hr. in a transwell. Cells invaded to the lower sides of the transwell, cells were fixed using 4% (w/v) paraformaldehyde and were stained using 0.05% crystal violet, and the cell number was counted as described before [28].

### Statistical analysis

McNemar test was used to determine the association between the expression of our gene of interest and the microRNA expression. Other data was analyzed using one-way Anova relative quantitation values were plotted using GraphPad Prism (La Jolla, CA). The observations were rendered statistically significant for P ≤ 0.05.

## Results

### miR-29 targets NMI

**Figure 1:**
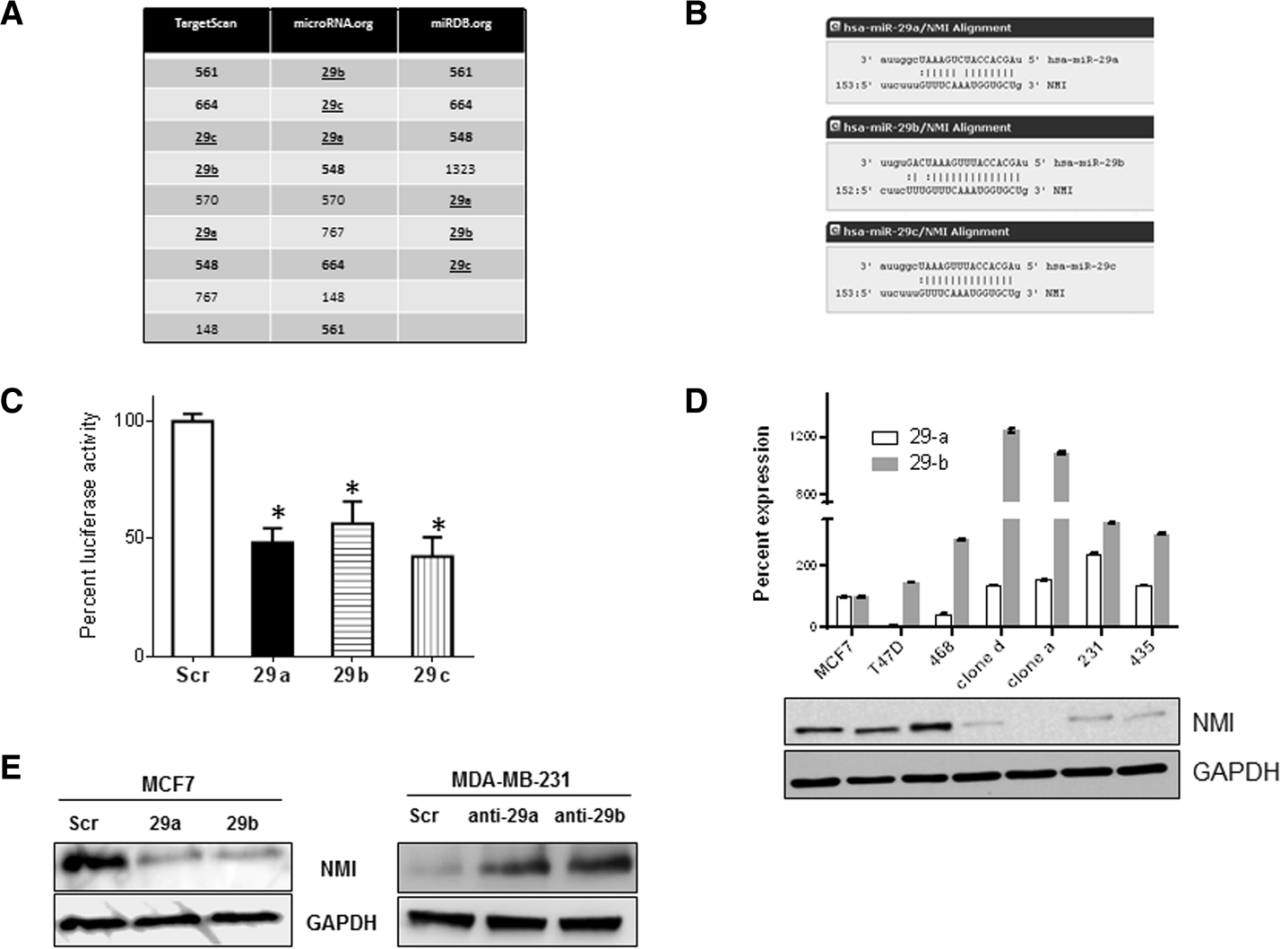
**miR-29 targets NMI** ***.*** **(A)** Micro-RNAs predicted to target the NMI transcript are catalogued using three different data bases represented as distinct columns. The top hits that were common to at least two data bases are represented. The highlighted miRNAs are common to all 3 searches. The miR-29 family is represented by underline. **(B)** Alignment of the miR-29 a, b, c seed sequence with the NMI 3′ UTR. Dashed lines represent complementary base pairing. **(C)** pMIR-REPORT29-NMI (containing putative binding site of miR-29 from the 3′ UTR of NMI) was co-transfected with pre-miR-29 or scrambled control. The assay was performed in triplicate and the experiment was performed twice. The luciferase activity readings were normalized with activity from a co-transfected β-gal expressing control. The error bars represent standard error of the mean (SEM). [*indicates p ≤0.05]. **(D)** Levels of each of the miR-29a and b mature transcripts from human breast cancer cell lines compared to MCF7, a breast cancer cell line with distinct epithelial like characters using real time-quantitive-PCR. Reactions were performed in triplicate. The corresponding level of NMI protein is seen in the adjacent western blot. GAPDH levels were used as loading control. **(E)** Transient transfection of MCF7 cells with pre-miR-29 a or b reduced NMI protein levels compared to scrambled control. On the other hand, MDA-MB-231 cells showed elevated levels of NMI upon introduction of anti-miR-29 a or b.

To identify candidate miRNAs that can potentially regulate NMI levels, we queried three publically available databases (Target Scan, microRNA.org and miRDB.org) and cataloged miRNAs predicted to target the NMI transcript (3′ UTR) (Figure 1A). The outputs of these searches were grouped to get the top hits, common to at least two databases. Six miRNAs *viz.* miR-561, miR-664, miR-548 and miR-29 a, b, c were commonly present among the three database searches. We investigated these miRNAs for their known relevance to cancer biology. We did not notice any significant cancer biology related publications for miR-561. miR-664 has a potential tumor suppressive activity in hepatocellular carcinoma and has been documented to downregulate methionine adenosyltransferase 1A (MAT1A) [29]. miR-548 is implied in regulating pancreatic cancer progression and downregulation of low-density lipoprotein receptor-related protein (LRP1B), in thyroid cancer [30, 31]. Our attention was specifically captured by miR-29 a, b, c because all the members of this family consistently ranked high in the prediction algorithms of all three databases searched. miR-29a is up-regulated and promotes self-renewal in AML and increases EMT and metastasis in breast cancer [32, 33]. miR-29b is induced by c-Myc in HMEC cells [34]. It is also upregulated in canine mammary cancer and multiple gene expression analyses indicate its upregulation in breast cancer [35–37]. miR-29a and c are also upregulated in drug resistant breast cancer [38]. Thus, based on our searches and compelling cues from published findings, we focused our studies on testing the ability of the miR-29 family in targeting NMI.miR-29 a, b, c target identical sequences in the NMI 3′ UTR with a slight alteration in their binding capacity based on variations outside the seed-sequence (Figure 1B). To validate targeting of the NMI 3′UTR by miR-29, we cloned the putative binding site of miR-29 in to the pMIR-REPORT™ vector to generate pMIR-REPORT29-NMI. This reporter was independently co-transfected with miR-29 a, b or c. We observed approximately 50% reduction in the activity of pMIR-REPORT29-NMI in MCF7 cells (Figure 1C). We did not observe any significant difference when activity of the miR-29 members was compared with each other, implying that they are capable of targeting NMI with comparable efficiency. Due to these similar abilities of the miR-29 family members, for this study we decided to focus on miR-29 a and b.

NMI expression is reduced in aggressive mesenchymal like cell lines [8]. We determined expression levels of miR-29 a and b in select cell types of reported epithelial or mesenchymal-like phenotype. MFC7, T47D and MDA-MB-468 are epithelial like cells whereas MCF10CA.cl.a, MCF10CA.cl.d, MDA-MB-231 are tumorigenic, highly invasive (mesenchymal-like) and metastatic cell lines. [MDA-MB-435 is considered as melanoma; however it is included in this panel as it has low levels of NMI, moreover NMI has been reported to reduce its invasive and tumorigenic potential [8, 9]]. We designated the observed levels of miR-29 a and b from MCF7 cells as a reference expression level in an epithelial-like cell line. We observed (Figure 1D) that the mesenchymal cells have notably elevated expression of miR-29 a and b compared to the epithelial-like MCF7 or T47D cells, implying a possible inverse relationship of NMI and miR-29 expression.To confirm the targeting of NMI by miR-29, we introduced miR-29 a or b in MCF7 cells by transient transfection. We observed that both miRNAs were capable of reducing the levels of NMI to a comparable extent. Conversely, inactivation of miR-29 would be expected to reinstate NMI expression. In MDA-MB-231 cells, silencing the expression of miR-29 a or b using respective anti-miRs elevated the level of NMI protein (Figure 1E). These observations confirm that NMI is targeted by miR-29 a and b in these cells.

### miR-29 expression enhances invasive ability

**Figure 2:**
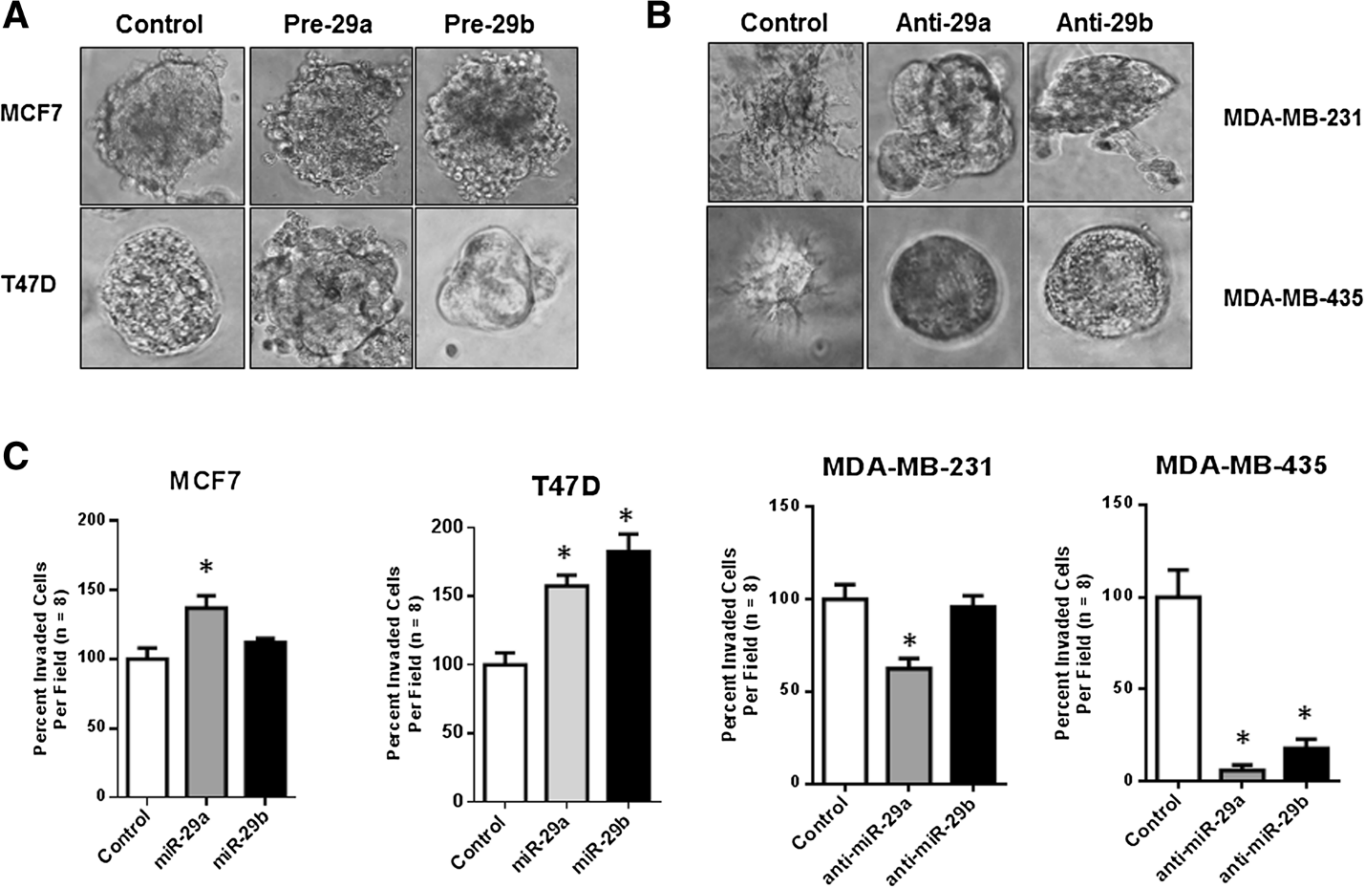
**miR-29a/b enhance mesenchymal phenotype. (A)** Breast cancer cells with significant epithelial phenotype MCF7 and T47D were treated with pre-miRNA HmiR0119-MR04 for 29a or HmiR0120-MR04 29b using transient transfection. The transfected cells were allowed to recover and were analyzed for their 3D structure. Fluorescence allowed for tracking only the colonies which grew from transfected cells. The 3D growth patterns were documented as photomicrographs using phase contrast microscopy. **(B)** Highly invasive breast cancer cells with noticeable mesenchymal phenotype MDA-MB-231 and Melanoma cells with highly invasive and mesenchymal phenotypes MDA-MB-435 were treated with anti-miRNA HmiR-AN0371-AM02 for 29a or HmiR-AN0373-AM02 for 29b using transient transfection. The transfected cells were allowed to recover and were analyzed for their 3D structure. Fluorescence allowed for tracking only the colonies which grew from transfected cells. The 3D growth patterns were documented as photomicrographs using phase contrast microscopy. **(C)** The same cells MCF7, T47D, MDA-MB-231 and MDA-MB-435 were transected by respective miRNA or antagomiRs as stated before and analyzed for their invasion using a modified Boyden chamber assay. The assay was performed for 16 hrs. Invaded cells were stained using crystal violet and enumerated. The results are represented as percent of control where the control is expressed as 100%. Error bars represent ± SEM and *indicates P ≤ 0.05

**Figure 3:**
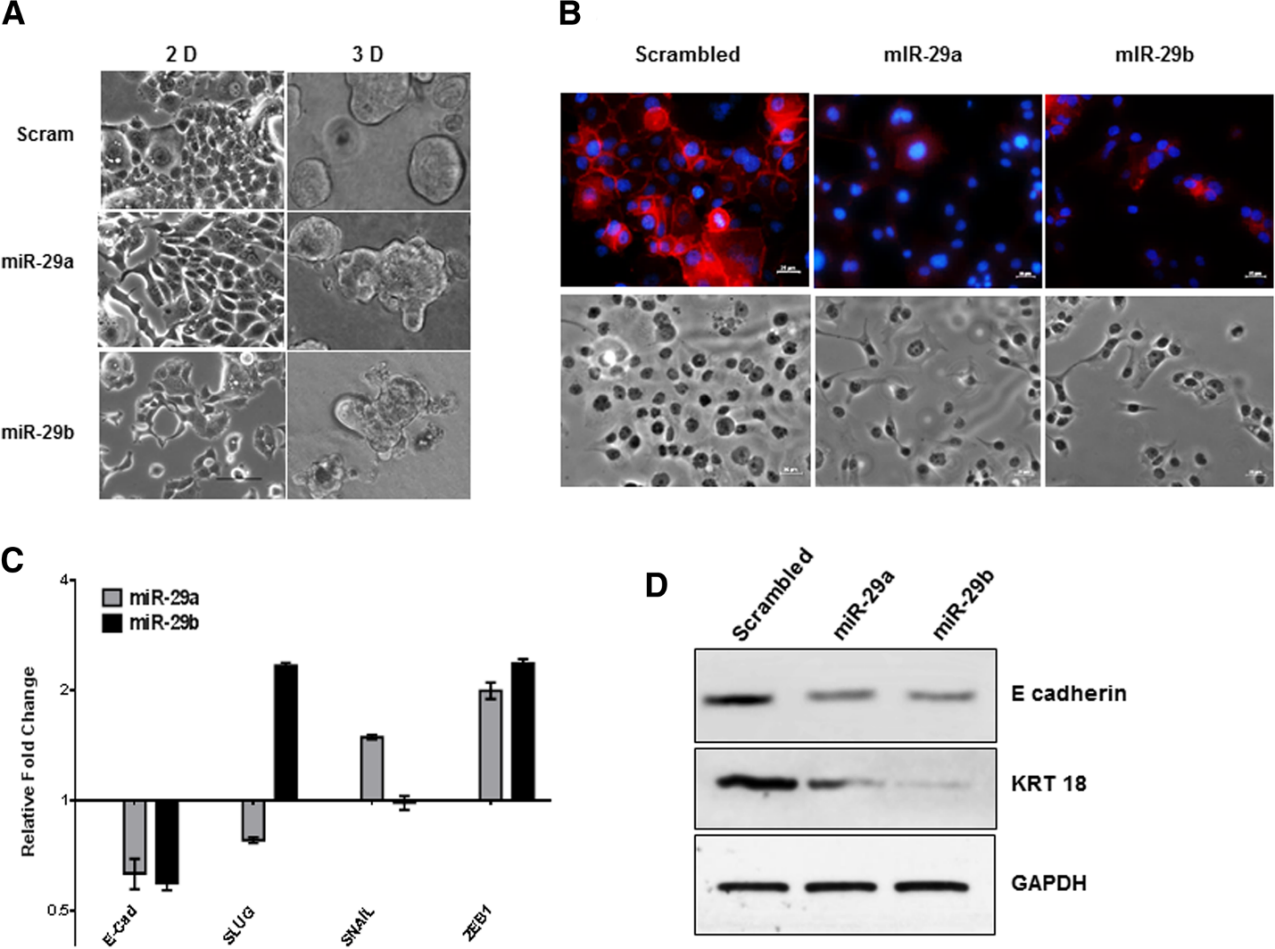
**miR-29 expression promotes mesenchymal plasticity. (A)** Growth morphologies of T47D cells stably expressing miR-29 a or b were evaluated in tissue culture plates (2D growth) or in matrigel (3D growth). The results were documented as photomicrographs using phase contrast microscopy. **(B)** T47D miR-29a and miR-29b cells show loss of E-cadherin staining at the membrane as compared to scrambled controls. E- cadherin (red), DAPI (blue), and phase contrast images were taken at 20× (Scale bar =25 μm). **(C)** Quantitative real-time PCR of markers for EMT, E-cadherin, Slug, Snail, or Zeb1 of T47D miR-29a or b expressing cells. Data are normalized to GAPDH expression and fold changes (log2) in expression are relative to corresponding scrambled control. **(D)** Protein levels of epithelial markers, E-cadherin and Keratin 18 (KRT18) are reduced by T47D miR-29a or b expression.

We have previously reported that NMI acts as one of the gatekeepers of the epithelial phenotype and our observations indicated that loss of NMI may be one of the key events preceding EMT [8]. This suggests a possible role of miR-29 in promoting invasion and EMT. To evaluate the functional impact of miR-29 a and b on EMT, we modulated (increased or decreased) expression of miR-29 in breast cancer cells with mesenchymal or epithelial-like phenotype and monitored their 3D growth morphology. MCF7 and T47D cells are epithelial like and form acinar structures in 3D growth matrix. Expression of miR-29a or b in these cells perturbed their growth morphology to grape-like or branched appearance. T47D cells were more responsive to shape change driven by miR-29a compared to miR-29b (Figure 2A). Conversely, when highly invasive MDA-MB-231 cells were evaluated for morphological changes after treatment with respective anti-miRs for miR-29 a or b, their 3D growth morphology lost its stellate appearance and acquired an irregular-spherical shape indicative of a shift toward acinar-like appearance. Similarly anti-miR treatments in MDA-MB-435 resulted in a stark shift to spheroid-like structures (Figure 2B). Additionally, the positive impact of miR-29 on invasive ability was evident when MCF7 and T47D transfected with miR-29 a and b showed noticeable increase in invasion (T47D: 150-200% and MCF7: 120-140%). Conversely, silencing miR-29 a or b from MDA-MB-435 cells dramatically reduced their invasive ability. In MDA-MB-231, silencing miR-29a led to a significant reduction in invasion whereas silencing miR-29b in these cells did not show a statistically significant effect (Figure 2C). Together, these observations indicate ability of miR-29 to promote invasive ability of breast cancer cells. However, it is interesting that the extent of reduction in invasive phenotype of MDA-MB-231 is much less striking than MDA-MB-435 though both are considered to be highly invasive. This suggests that there are complex intrinsic molecular underpinnings that decide invasive properties and these may vary for each cell line. Thus their response to a single molecular alteration may reveal only a partial picture.We established T47D derived cell lines stably expressing miR-29a and miR-29b. The growth morphology of the miR-29 expressors looked noticeably different when adherent growth on tissue culture plates was examined. Unlike the scrambled control that retained the epithelial-like cobble stone morphology with well-defined cell-to-cell contacts, the miR-29 expressors showed an irregular fibroblastic appearance with cells that did not grow as tightly packed structures but kept distinct separation from adjacent cells (Figure 3A and B). The 3D growth morphology of these cells showed a grape like and protrusive patterns unlike the control (Figure 3A). At the molecular level, these cells showed lack of E-cadherin staining at the cell-cell junctions (Figure 3B). This loss was noted at transcript levels as well as confirmed at the protein level by western blot analysis (Figure 3D). Concomitant loss of keratin 18 was also observed supporting a shift to a more mesenchymal-like molecular profile. Interestingly, a loss of KRT18 was not noticed at the transcript level but we did notice a concomitant gain of mesenchymal transcription factors SNAIL and ZEB1 in T47D cells expressing miR-29 a and SLUG and ZEB1 in miR-29b expressors. Thus, though the resultant impacts of miR-29a or b on specific molecular indicators of EMT are not identical, overall our observations indicated a clear induction of a mesenchymal plasticity following miR-29 expression.

### miR-29 and NMI levels show inverse trends in breast cancer tissues

**Figure 4:**
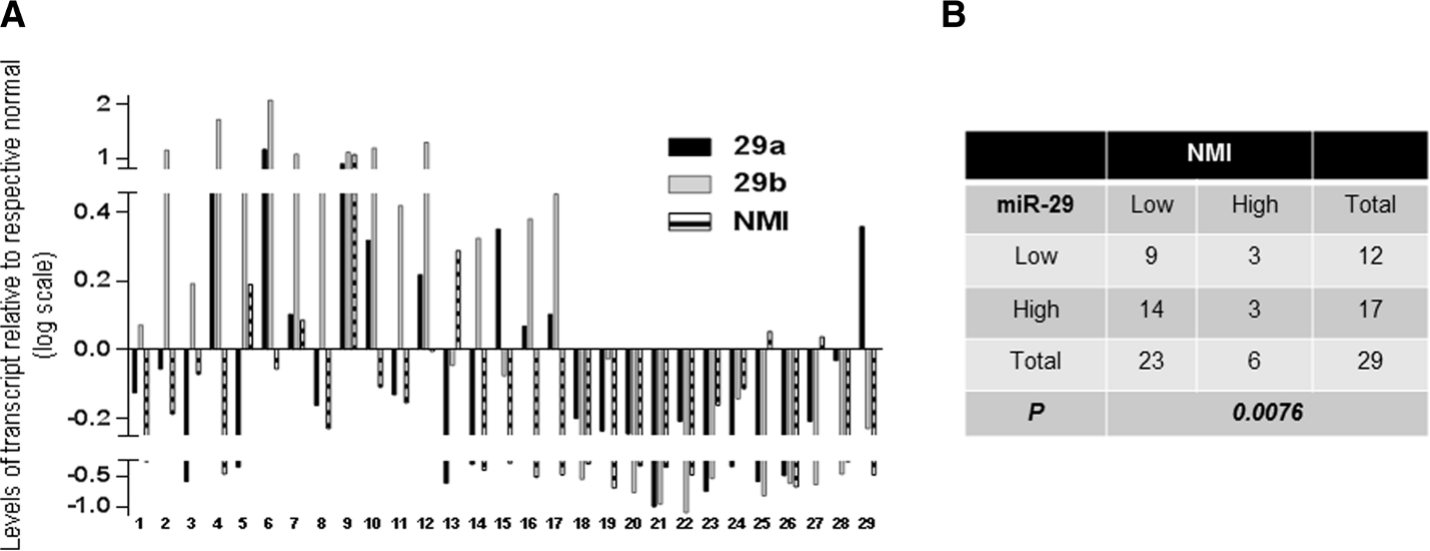
**Inverse trend of expression for NMI and miR-29. (A)** Total RNA from a panel of fresh frozen 29 invasive breast tumor specimens with matching normal tissue was analyzed by qRT-PCR for the levels of miR-29 a, b and NMI. The values were normalized with individual surrounding un-involved tissue. The results are expressed as level of transcript compared to individual un-involved tissue. **(B)** Association between the expression of NMI and the microRNA expression was determined using McNemar’s test. High indicates positive value compared with respective uninvolved tissue. Low indicates negative value compared to respective uninvolved tissue.

To determine the relationship of miR-29 levels with NMI expression in patient derived specimens, we analyzed total RNA from a panel of fresh frozen 29 breast tumors (that comprised primaries from patients with Stage III and IV disease) and compared with matching surrounding (un-involved) tissue by qRT-PCR for the levels of miR-29 a, b and NMI. We observed a decrease in NMI expression in 24 out of 29 specimens. miR-29 a or b or both were upregulated in 17 of the total 29 (Figure 4A). McNemar’s test was used to determine the association between the expression of NMI and miR-29 a and b expression. Up-regulation of either miR-29 a or b (or both) was considered a contributing event toward impacting NMI levels. As depicted in Figure 4B a strong, inverse relationship between the reduced expression of NMI and miR-29 expression was observed (P < 0.0076). Furthermore, 14 of 17 patients with high miR-29 expression showed low NMI expression and in three patients with high NMI expression low expression of miR-29 was noted (p < 0.01). These observations provide convincing in-vivo evidence of an inverse relationship between NMI and miR-29.

### Absence of NMI expression positively influences miR-29 levels

**Figure 5:**
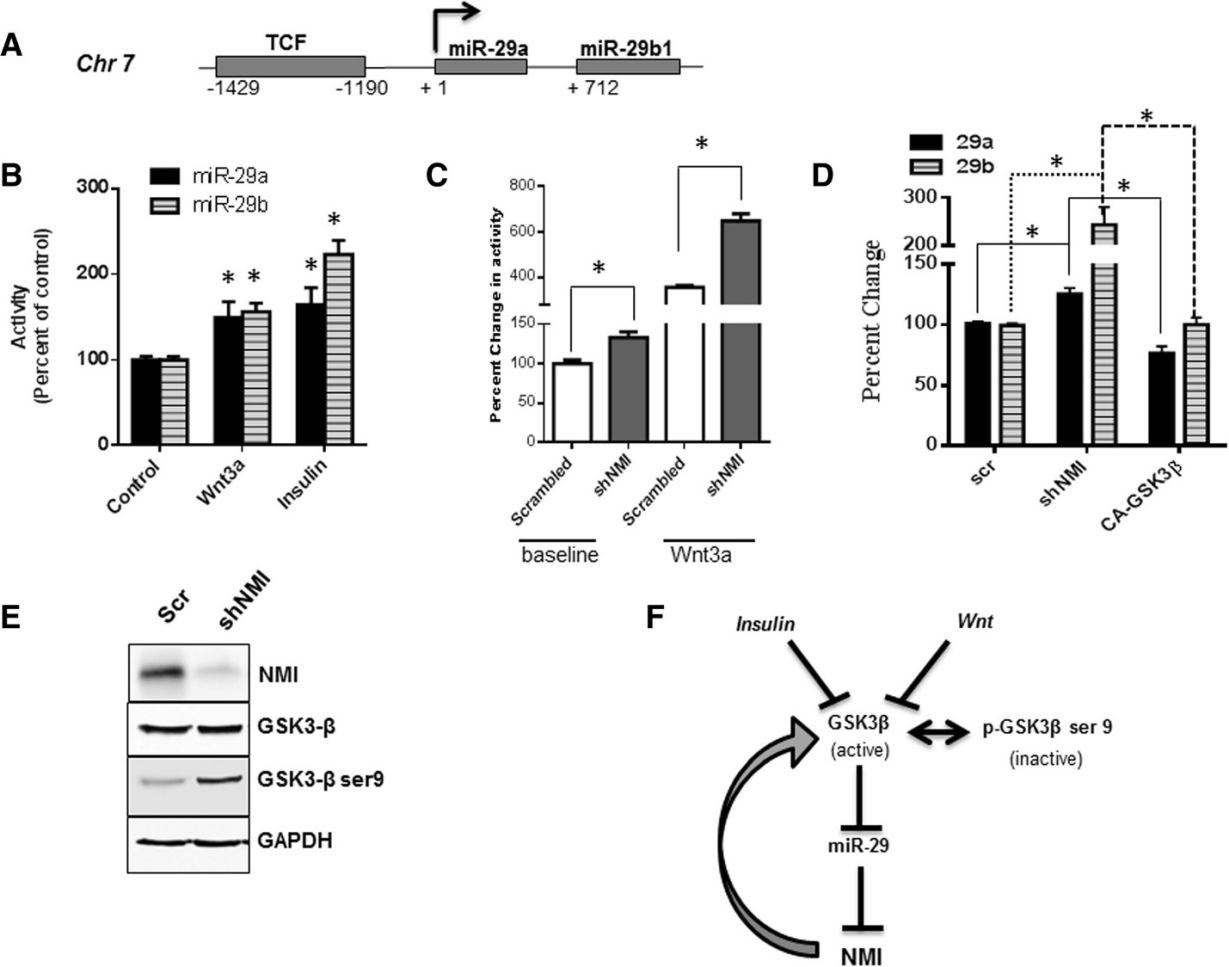
**Upregulation of miR-29 as a consequence of compromised NMI expression. (A)** ChIP-data reported by the ENCODE project confirms the existence of a TCF site in the upstream regulatory region of miR-29 a and b1 on chromosome 7. **(B)** T47D cells grown in growth factor free medium were treated with Wnt3a ligand (100 ng/ml) or insulin (10 μg/ml) for 24 hr. Levels of miR-29 a/b using RT-PCR. The levels were compared to untreated cells (control), set arbitrarily at 100%. **(C)** T47D-shNMI cells (stably silenced for NMI) were evaluated for β-catenin driven transcription activity (using TOP-FLASH reporter) at basal levels or with Wnt3a (100 ng/ml) treatment. This activity was compared to the T47D-scrambled control cells. Error bars represent ± SEM and *indicates P ≤ 0.05. **(D)** Total RNA from T47D-shNMI cells was analyzed for levels of miR-29a and b and compared with T47D-scrambled control cells. Introduction of constitutively active GSK3β (CA-GSK3β) in T47D-shNMI resulted in significant reduction of miR-29 expression. Error bars represent ± SEM and *indicates P ≤ 0.05. **(E)** Total protein from T47D-shNMI and corresponding scrambled control cells was analyzed for the levels of total GSK3β and phospho-Ser-9-GSK3β using western blot analysis. Level of NMI confirmed the stable silencing of NMI in these cells and GAPDH was used as a loading control. **(F)** Proposed model of miR-29 signaling: In absence of NMI and/or due to micro-environmental signals such as Wnt or insulin, GSK3β is inactivated (upon phosphorylation at Ser 9). That leads to increased miR-29 levels possibly by elimination of check on TCF driven transcription. miR-29 in turn targets NMI, which is one of the factors responsible to guard the active status of GSK3β; thus driving a feed forward loop for miR-29 activation and sustained loss of NMI. This signaling mechanism may be one of the promoters of invasive progression of breast cancer.

Studies in human osteoblasts have indicated that Wnt signaling positively regulates miR-29 expression [39, 40]. We queried the ENCODE ChIP-Seq database and found that the miR-29 a/b1 gene is a target of TCF transcription factor(s) (Figure 5A) [41–43]. To test the impact of Wnt on miR-29 in breast cancer cells, we treated T47D cells independently with Wnt3a or insulin and found a noticeable increase in levels of miR-29 a and b (Figure 5B). Insulin and Wnt actions result in eventual inactivation of GSK3β status. Our previous findings had indicated that overexpression of NMI can negatively impact Wnt/β-catenin signaling [9]. We analyzed activity of a Wnt/β-catenin reporter (TOP-FLASH) in T47D cells stably silenced for NMI expression (T47D-shNMI) [8]. Compared to the scrambled control, these cells showed higher baseline activity of TOP-FLASH. Consistent with our expectation, Wnt3a stimulus showed a robust activation of the reporter in T47D-shNMI cells (Figure 5C). These findings indicate that silencing NMI expression results in reduced constraints on canonical Wnt signaling. These cells also showed significant elevation of miR-29 a and b (Figure 5D). Both Wnt and insulin signaling pathways commonly involve the inactivation of the multifunctional kinase GSK3β. NMI silenced cells show increased phospho-Ser-9-GSK3β (Figure 5E). Phospho-Ser-9-GSK3β is an inactive form of GSK3β that is incapable of restricting canonical Wnt signaling. This inactivation may consequently result in elevated miR-29 expression. Introduction of constitutively active GSK3β (GSK3β-S9A) in T47D-shNMI resulted in significant reduction of miR-29 expression (Figure 5D). This indicates that inactivation of GSK3β due to absence of NMI results in upregulation of miR-29 expression. Overall this data suggests that loss of NMI will result in feed-forward activation of miR-29 expression.

## Conclusion

Loss of NMI during breast cancer progression promotes invasion and metastasis [8]. However, the reasons behind this loss of expression were unclear. As summarized in Figure 5F, our observations provide the first clue that miR-29a and miR-29b are involved in negatively regulating the expression of NMI in breast cancer. Breast cancer lacks classical Wnt activating mutations (such as APC) that are seen in colon cancer. However, aberrant Wnt response in the form of nuclear β-catenin is frequently observed in breast cancer [44–47]. In the tumor microenvironment Wnt ligands are known to be present. As well as alternative signaling that inactivates GSK3β or suppressors of Wnt inhibitors (such as SFRPs, Dickkopf proteins or Wif1) can aberrantly alter Wnt homeostasis to an activated state [48]. Thus, the increased miR-29 levels due to micro-environmental signals and/or as result of the feed forward effect of reduced NMI will prompt additional lowering of NMI levels and consequently result in unrestricted β-catenin signaling. The Wnt/β-catenin pathway has a fundamental impact on tumor progression including EMT and cancer stem cell phenotype. Thus molecular alterations that contribute to the dysregulation of Wnt homeostasis are critical to understanding intricate details of the invasive progression of breast cancer. miRNAs are mediators of the morphogen gradient during development and thus the role of miRNAs in EMT is anticipated [49].

Diverse roles have been attributed to miR-29. It has been shown as a tumor suppressor due to its involvement in promoting tumor cell apoptosis, by suppressing DNA methylation of tumor-suppressor genes leading to reduced proliferation and increased chemosensitivity [50]. Additionally in cells with wild type P53, miR-29 expression supports stabilization of P53 [51]. However, its role in EMT is debatable. Reports by Chou *et al.* imply that loss of miR-29b is an EMT and metastasis promoting event [52]. Contrary to that and consistent with our observations, Gebeshuber *et al.* have shown that miR-29a promotes EMT and metastatic ability by targeting tristetraprolin [33]. Wang *et al.* have shown that miR-29b expression promoted migration and invasion of human breast cancer cells possibly due to its ability to target the tumor suppressor PTEN [53]. A notable observation regarding the biologic role of miR-29 is that transgenic miR-29 overexpressing mice spontaneously developed indolent chronic lymphocytic leukemias [54]. The reasons for these diverse roles of miR-29 could be many. Individual miRNAs molecules target a wide range of mRNAs. Thus, their role in cancer is highly context-dependent. It depends on the expression status of their target mRNAs, which vary based on cell types. Additionally, within a cell type, there may be temporal differences that may be dictated by cell physiology and impact of tumor microenvironment. MiR-29a and miR-29b1 are transcribed from a locus on chromosome 7 while the miR-29b2 and miR-29c locus is on chromosome 1. Based on their promoter sequence, both loci have distinct regulatory elements. The existence of such complex distinct regulation for the miR-29 family members is a compelling indicator of context-dependent regulation of these miRNAs. Consequently diverse impacts on their target genes and resultant diverse phenotypic manifestations are conceivable. Our analysis of RNA from tumors specimens corresponding to patients with invasive and metastatic stage breast cancer shows an inverse relationship miR-29 and NMI and thus underscores the disease relevance of our findings. Cumulatively, we conclude that miR-29 family has a critical role in mediating loss of NMI and subsequent EMT in breast cancer progression.
